# Incarvillateine produces antinociceptive and motor suppressive effects via adenosine receptor activation

**DOI:** 10.1371/journal.pone.0218619

**Published:** 2019-06-25

**Authors:** Jinwoo Kim, Diane M. Bogdan, Matthew W. Elmes, Monaf Awwa, Su Yan, Joyce Che, Garam Lee, Dale G. Deutsch, Robert C. Rizzo, Martin Kaczocha, Iwao Ojima

**Affiliations:** 1 Department of Chemistry, Stony Brook University, Stony Brook, New York, United States of America; 2 Institute of Chemical Biology and Drug Discovery, Stony Brook University, Stony Brook, New York, United States of America; 3 Department of Anesthesiology, Stony Brook University, Stony Brook, New York, United States of America; 4 Department of Biochemistry and Cell Biology, Stony Brook University, Stony Brook, New York, United States of America; 5 Department of Applied Mathematics and Statistics, Stony Brook University, Stony Brook, New York, United States of America; University of Arizona College of Medicine, UNITED STATES

## Abstract

(-)-Incarvillateine (INCA) is a natural product that has garnered attention due to its purported analgesic effects and historical use as a pain reliever in China. α-Truxillic acid monoesters (TAMEs) constitute a class of inhibitors targeting fatty acid binding protein 5 (FABP5), whose inhibition produces analgesia in animal models. The structural similarity between INCA and TAMEs motivated us to assess whether INCA exerts its antinociceptive effects via FABP inhibition. We found that, in contrast to TAMEs, INCA did not exhibit meaningful binding affinities toward four human FABP isoforms (FABP3, FABP4, FABP5 and FABP7) *in vitro*. INCA-TAME, a putative monoester metabolite of INCA that closely resembles TAMEs also lacked affinity for FABPs. Administration of INCA to mice produced potent antinociceptive effects while INCA-TAME was without effect. Surprisingly, INCA also potently suppressed locomotor activity at the same dose that produces antinociception. The motor suppressive effects of INCA were reversed by the adenosine A_2_ receptor antagonist 3,7-dimethyl-1-propargylxanthine. Collectively, our results indicate that INCA and INCA-TAME do not inhibit FABPs and that INCA exerts potent antinociceptive and motor suppressive effects at equivalent doses. Therefore, the observed antinociceptive effects of INCA should be interpreted with caution.

## Introduction

Chronic pain is a major worldwide healthcare issue today. The annual economic costs of pain are estimated to be $560–635 billion, and carry even higher costs in terms of human suffering and impact on quality of life [1]. Alongside the increased medical costs of pain management, major pain relievers such as opioids, antidepressants, and nonsteroidal anti-inflammatory drugs (NSAIDs) suffer from their own drawbacks including low efficacy and/or untoward side-effects, including overdose related death [2, 3]. Thus, new classes of pain killers with alternative mechanisms of action (MOA) are highly desired. *Incarvillea sinensis* (from the Bignoniaceae plant family) has garnered interest due to its reported analgesic actions [4]. The dried plant matter (known as ‘Jiao Hao’ or ‘Cheron’) has been used as an herbal pain remedy for millennia in traditional Eastern medicine. (-)-Incarvillateine (INCA), a monoterpene alkaloid produced by the plant, is considered to be the major active component [5]. However, INCA’s MOA has not yet been conclusively established, with previous work suggesting the possible involvement of several receptor systems [4, 6, 7]. An incipient study suggested an interaction with the central opioid system because INCA’s antinociceptive effects were partially reversed by the opioid receptor antagonist naloxone in formalin-induced rodent pain models, though the authors state that the MOA is different from that of morphine whose analgesic effects were completely reversed by the same antagonist [6]. More recently, the adenosine A_1_ receptor was suggested as the major target mediating INCA’s antinociceptive effects [4].

**Fig 1:**
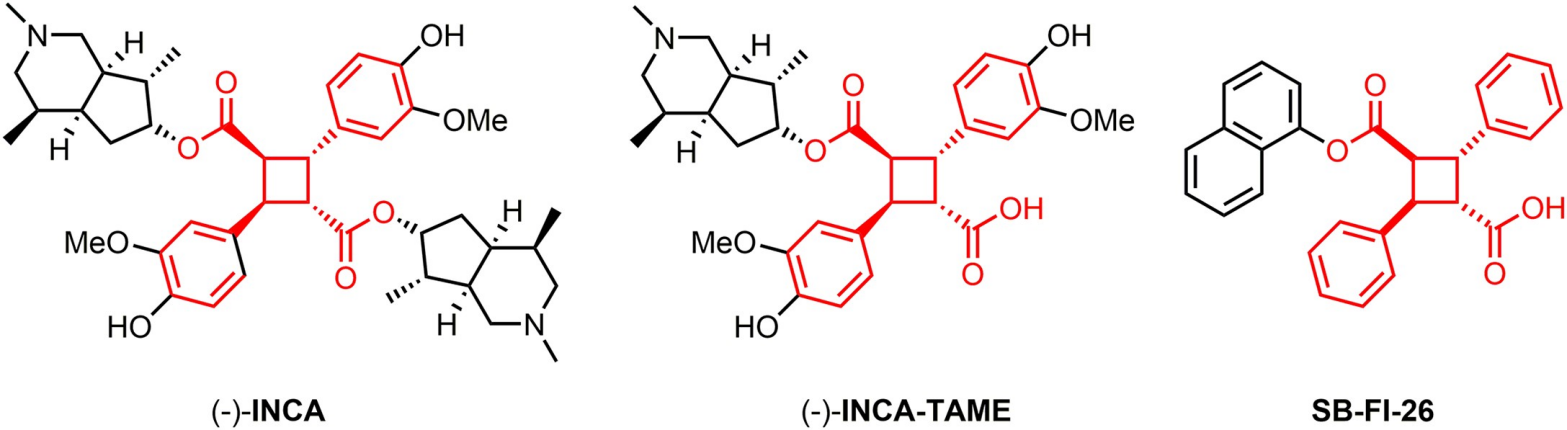
Structures of INCA and INCA-TAME.

A previous drug discovery study by our laboratories employed high-throughput virtual screening to identify novel antinociceptive compounds targeting the fatty acid binding proteins (FABPs) and found that a lead compound, SB-FI-26, shares the same cyclobutanedicarboxylate skeleton, i.e., truxillate moiety, with INCA [8] (Fig 1). The structural similarity and antinociceptive activities of INCA and SB-FI-26 has led us to hypothesize that inhibition of FABPs may underlie, wholly or in part, the MOA of INCA. Additionally, considering the fact that SB-FI-26 is a monoester while INCA is a diester, it is possible that INCA may be converted to the corresponding monoester through biotransformation *in vivo*. To better elucidate the MOA of this natural antinociceptive compound, INCA and its monoester (INCA-TAME) were synthesized in house. The compounds were tested for binding to FABPs *in vitro* and in a mouse model of pain and locomotion. Our results indicate that INCA and INCA-TAME do not target FABPs and further show that INCA exerts potent motor suppressive effects at doses that produce analgesia.

## Materials and methods

### Ethics statement

The experiments conducted herein were approved by the Stony Brook University institutional animal care and use committee (#277150).

### Chemicals

Dimethylsulfoxide (DMSO) and Cremophor-EL were from Sigma. 12-*N*-methyl-(7-nitrobenz-2-oxa-1,3-diazo)aminostearic acid (NBD-stearate) was purchased from Avanti Polar Lipids (Alabaster, AL, USA). 1-Anilinonaphthalene-8-sulfonic acid (ANS) were purchased from Cayman Chemical Company (Ann Arbor, MI, USA). All reagents and solvents used were of the highest grade commercially available.

### Synthesis of INCA and INCA-TAME

#### Synthesis of (-)-Incarvilline (1)

Following previously published synthetic routes [9], a commercially available (*R*)-(-)-carvone (15.5 g) was used as a starting material to give (-)-incarvilline (**1**) (872 mg, 4.6% overall yield for 11 steps) as a white solid: m.p. 92–94 °C (lit. [10] 94.4–95.5 °C) [α]^21^D –8.51 (*c* 0.47, CHCl_3_) (lit. [9] [α]^23^D –8.9 (*c* 0.75, CHCl_3_)); ^1^H NMR (700 MHz, CDCl_3_) δ 4.28 (t, *J* = 5.9 Hz, 1H), 2.64 (dd, *J* = 11.0, 6.0 Hz, 1H), 2.48 (dd, *J* = 11.0, 3.4 Hz, 1H), 2.40–2.36 (m, 1H), 2.20 (s, 3H), 2.08–2.02 (m, 1H), 1.90–1.86 (m, 1H), 1.81–1.76 (m, 2H), 1.64 (t, *J* = 11.5 Hz, 1H), 1.53 (t, *J* = 11.7 Hz, 1H), 1.47 (dd, *J* = 13.6, 7.2 Hz, 1H), 0.99 (d, *J* = 7.4 Hz, 3H), 0.84 (d, *J* = 7.0 Hz, 3H); ^13^C NMR (150 MHz, CDCl_3_) δ 73.3, 58.12, 58.10, 46.4, 45.9, 42.4, 37.5, 32.7, 30.6, 17.5, 14.3; HRMS (ESI-TOF) *m/z* calcd for C_11_H_22_NO [M+H]^+^ 184.1623, found 184.1699. Data are consistent with the literature values [9, 10].

#### Synthesis of (-)-Incarvillateine (INCA)

To a solution of tosylated α-truxillic acid (**2**) (0.61 g, 0.87 mmol) prepared as reported in literature [9, 10] in anhydrous tetrahydrofuran (THF, 20 mL) were added pivaloyl chloride (0.24 mL, 1.92 mmol) and trimethylamine (0.27 mL, 1.92 mmol) at -78 °C. Then, the mixture was warmed to 0 °C with stirring for 1h. The reaction mixture was filtered, washed with THF and concentrated to dryness. After dissolving the crude product in acetonitrile (10 mL), (-)-incarvilline (**1**) (0.32 g, 1.73 mmol) and 4-dimethylaminopyridine (DMAP) (0.02 g, 0.21 mmol) were added and the reaction mixture was stirred for 3 days at room temperature. After removal of the solvent under reduced pressure, the residue was purified by flash chromatography on silica gel (CH_2_Cl_2_/MeOH/NH_4_OH = 100:10:1) to afford tosylated (-)-incarvillateine (511 mg, 57%, 2 steps): ^1^H NMR (300 MHz, CDCl_3_) δ 7.76 (d, *J* = 7.8 Hz, 4H), 7.32 (d, *J* = 7.8 Hz, 4H), 7.07 (dd, *J* = 8.0, 3.6 Hz, 2H), 6.84–6.75 (m, 4H), 4.83–4.81 (m, 2H), 4.41–4.30 (m, 2H), 3.88–3.78 (m, 2H), 3.62 (s, 3H), 3.60 (s, 3H), 2.66–2.61 (m, 2H), 2.54–2.49 (m, 2H), 2.46 (s, 6H), 2.22 (s, 6H), 2.04–1.97 (m, 2H), 1.89–1.84 (m, 4H), 1.78–1.58 (m, 6H), 1.54–1.44 (m, 3H), 1.20–1.13 (m, 1H), 0.79–0.73 (m, 9H), 0.57 (d, *J* = 7.3 Hz, 3H).

The removal of the tosyl groups was carried out by following Kibayashi’s procedure [10]. To a solution of tosylated (-)-incarvillateine (0.39 g, 0.37 mmol) in methanol (MeOH, 37 mL), sodium/amalgam (20%, 1.29 g) was added at room temperature and the reaction mixture was stirred for 4 h. After quenched with sat. NH_4_Cl solution and extracted with CH_2_Cl_2_, the organic layer was dried over MgSO_4_, filtered, and concentrated in vacuo. The residue was purified by flash chromatography on silica gel (CH_2_Cl_2_/MeOH/NH_4_OH = 100:10:1) and dried *in vacuo* to afford (-)-incarvillateine (227 mg, 84%) as a white solid: m.p. 216–218 °C (lit. [10] 217–218 °C); [α]^21^D –12.5 (*c* 0.48, CHCl_3_)) (lit. [9] [α]^23^D –14.8 (*c* 1.39, CHCl_3_); lit. [10] [α]^20^_D_ –10.9 (c 0.06, CHCl_3_)); ^1^H NMR (700 MHz, CDCl_3_) ^1^H NMR (700 MHz, CDCl_3_) δ 6.84–6.77 (m, 6H), 4.91–4.84 (m, 2H), 4.90 (t, *J* = 6.5 Hz, 1H), 4.85 (t,*J* = 6.5 Hz, 1H), 4.36–4.34 (m, 1H), 4.31–4.29 (m, 1H), 3.89 (s, 3H), 3.87 (s, 3H), 3.84–3.80 (m, 2H), 2.57–2.56 (m, 2H), 2.46–2.45 (m, 2H), 2.19 (s, 6H), 2.14–2.09 (m, 1H), 1.98–1.95 (m, 3H), 1.84–1.82 (m, 3H), 1.72–1.66 (m, 2H), 1.61–1.56 (m, 2H), 1.55–1.51 (m, 1H), 1.43 (t, *J* = 10.0 Hz, 2H), 1.05 (dd,*J* = 13.7, 8.0 Hz, 1H), 0.80 (d, *J* = 7.3 Hz, 3H), 0.75 (d, *J* = 6.7 Hz, 3H), 0.70 (d, *J* = 6.7 Hz, 3H), 0.60 (d, *J* = 7.3 Hz, 3H), 0.54 (dd, *J* = 13.7, 8.0 Hz, 1H); ^13^C NMR (175 MHz, CDCl_3_) δ 172.1, 171.8, 146.9, 146.8, 145.4, 145.3, 130.7, 130.6, 120.5, 120.0, 114.7, 111.0, 110.9, 76.6, 76.4, 57.4, 57.3, 57.1, 57.0, 56.0, 55.9, 48.0, 47.4, 46.0, 45.9, 45.7, 45.6, 41.9, 41.3, 40.5, 40.4, 37.4, 37.3, 30.2, 30.1, 29.8, 29.3, 17.2, 17.0, 15.0, 14.5; HRMS (ESI-TOF) *m/z* calcd for C_42_H_59_N_2_O_8_ [M+H]^+^ 719.4193, found 719.4199. Data are consistent with the literature values [9, 10].

#### Synthesis of (-)-Incarvillateine monoester (INCA-TAME)

**Fig 2:**
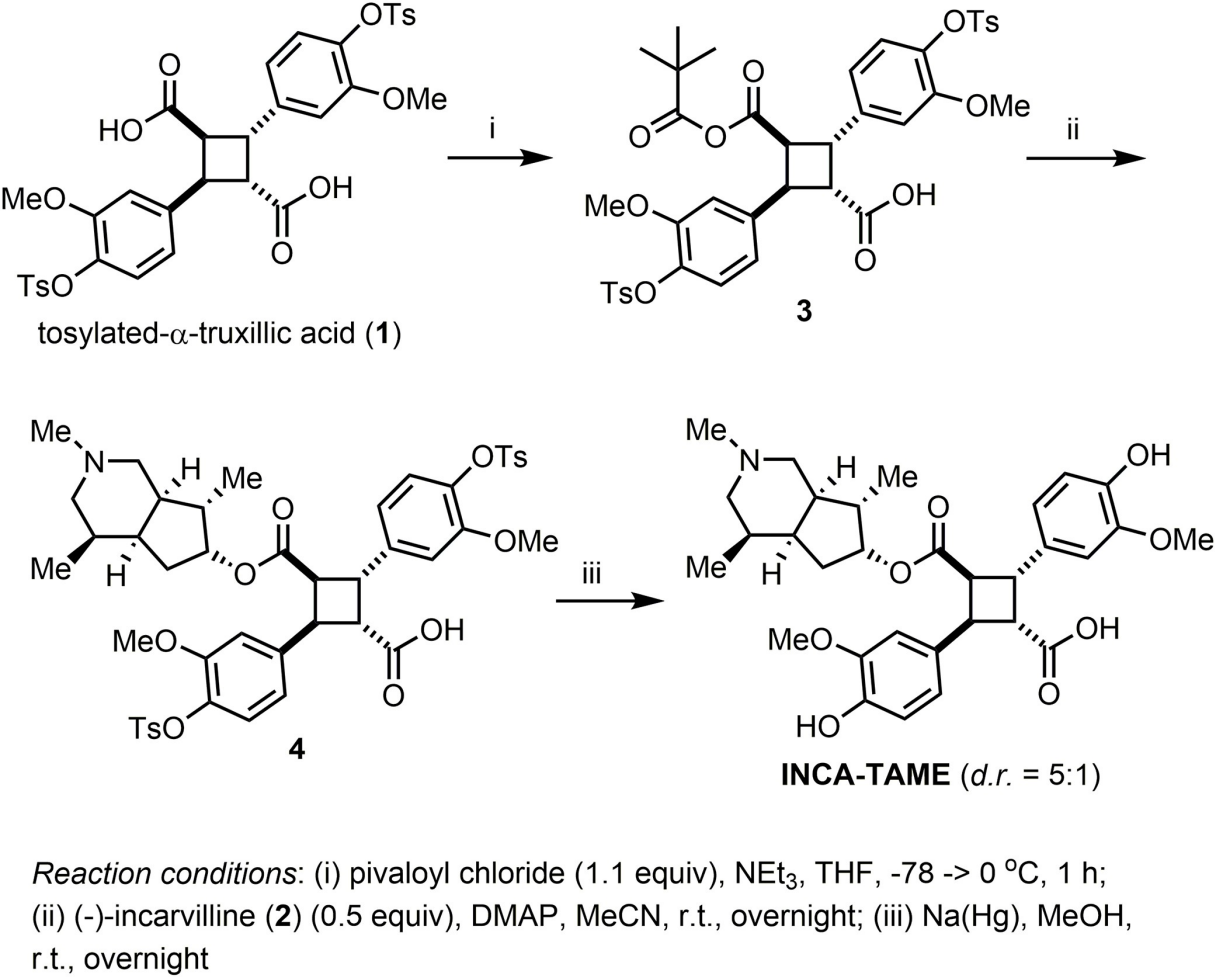
Synthesis of INCA-TAME.

INCA-TAME was synthesized through monoesterification of tosylated α-truxillic acid (**2**) with (-)-incarvilline (**1**), followed by removal of the tosyl group (Fig 2). To a solution of **2** (0.30 g, 0.44 mmol) in anhydrous THF (10 mL), pivaloyl chloride (0.06 mL, 0.48 mmol) and triethylamine (0.07 mL, 0.48 mmol) were added at -78 °C, and the reaction mixture was stirred at 0 °C for 1 h. After filtration and washing with THF, the reaction mixture was concentrated under reduced pressure to give mixed anhydride **3**. Then, **3** was dissolved in acetonitrile and **1** (0.04 g, 0.22 mmol) and DMAP (0.01 g, 0.10 mmol) were added. The reaction mixture was stirred at room temperature overnight. After the removal of the solvent *in vacuo*, the residue was purified by flash chromatography on silica gel (CH_2_Cl_2_/MeOH/NH_4_OH = 100:10:1) to give the corresponding monoester **4**. Compound **4** was dissolved in MeOH (9.9 mL), and sodium/amalgam (0.33 g, 20%) was added to the solution. The reaction mixture was stirred at room temperature overnight. After the solvent was removed *in vacuo*, the residue was purified by flash chromatography on a C18-reversed phase silica gel (Sigma-Aldrich, 230–400 mesh) (MeOH/H_2_O = 50:50 with 0.1% trifluoroacetic acid) to afford (-)-incarvillateine monoester (INCA-TAME) (18 mg, 7% for 3 steps) as a light brown oil (5:1 diastereomer mixture by ^1^H NMR); [α]^22^D – 3.70 (*c* 0.54, MeOH); ^1^H NMR (700 MHz, acetone-d_6_) δ 7.75 (d, *J* = 7.2 Hz, 1H), 7.19 (d, *J* = 7.2 Hz, 1H), 7.04 (s, 1H), 7.0 (s, 1H), 6.86–6.84 (m, 2H), 6.80 (d, *J* = 8.0 Hz, 1H), 4.95–4.94 (m, 1H), 4.40–4.38 (m, 2H), 4.00 (t, *J* = 8.7 Hz, 1H), 3.89 (s, 3H), 3.87 (s, 3H), 3.35 (s, 2H), 3.23–3.22 (m, 1H), 2.73 (m, 1H), 2.59 (m, 1H), 2.36 (s, 3H), 2.32–2.31 (m, 1H), 1.92–1.90 (m, 1H), 1.89–1.85 (m, 1H), 1.31 (s, 1H), 1.19 (dd, *J* = 13.5, 7.7 Hz, 1H), 0.89 (d, *J* = 6.7 Hz, 3H), 0.80 (d, *J* = 7.0 Hz, 3H); ^13^C NMR (175 MHz, acetone-d_6_) δ 173.7, 172.1, 162.1, 148.2, 148.1, 146.5, 146.4, 140.8, 131.84, 131.79, 129.3, 127.2, 121.2, 120.9, 115.6, 115.5, 112.53, 112.51, 76.0, 56.4, 56.3, 55.1, 54.4, 48.8, 47.7, 44.3, 43.7, 42.5, 42.1, 41.1, 37.3, 21.3, 16.7, 14.9; HRMS (ESI) *m/z* calcd for C_31_H_40_NO_8_ [M+H]^+^ 554.2675, found 554.2747.

### FABP purification

Human FABP3, FABP4, FABP5, and FABP7 were cloned into pET28a vectors, transformed into BL21(DE3) *E*. *coli*, and purified by nickel-affinity chromatography exactly as previously described [8]. Any residual endogenous bacterial lipids were removed from the proteins by incubation in a column of hydroxyalkoxypropyl-dextran (Sigma Chemical Co., St Louis, MO, USA) for 1 hour at 37°C with occasional shaking.

### FABP binding assays

In vitro fluorescence displacement binding assays were carried out in 96-well Costar plates (Corning Life Science, Kennebunk, ME, USA). Recombinant human FABP (3 μM) was incubated with NBD-stearate or ANS (500 nM) in binding assay buffer (30 mM Tris-HCl, 100 mM NaCl, pH 7.6). Competitor test compounds (INCA and INCA-TAME, 5–50 μM) were then introduced to the well, mixed, and the system was allowed to equilibrate for 20 minutes at 25°C in the dark. All experimental conditions were tested in triplicate. Each independent assay included a strong competitive binder (arachidonic acid, 10 μM) as a positive control for FABP-bound probe displacement. Loss of fluorescence intensity was monitored with an F5 Filtermax Multi-Mode Microplate Reader (Molecular Devices, Sunnyvale, CA, USA) using excitation (ex.) and emission (em.) wavelengths appropriate for each probe (NBD-stearate ex./em. = 465/535 nm, ANS ex./em. = 370/470 nm). Following background subtraction, the raw fluorescence intensity values were normalized and visualized using the Graphpad Prism software (Prism version 7.0 for Mac OS: Graphpad software Incorporated, La Jolla, CA, USA)

### Behavioral assays

Thermal paw withdrawal and locomotor activity assays were performed as previously described [8, 11, 12]. For the pain assays, male mice received an intraplantar injection of complete Freund’s adjuvant (CFA) and paw withdrawal latencies were assessed using a Hargreaves apparatus (Ugo Basile). INCA (10 mg/kg), INCA-TAME (20 mg/kg), or vehicle (1:1:8, DMSO:Cremophor:Saline) were injected via the intraperitoneal route in a volume of 10 μl/g body weight and latencies were assessed 90 min later. Locomotor activity during the dark phase was examined using the PAS home cage apparatus (San Diego Instruments). Inhibitors or vehicle were injected 15 min before the dark phase and locomotor activity was measured for 6 h.

### Statistical analysis

Thermal withdrawal latencies at baseline and after CFA as well as locomotor activity were assessed using One-way or Two-way ANOVA followed by Dunnett’s or Tukey posthoc test as appropriate. We consider a p-value less than 0.05 as statistically significant.

### Computational assessment

Prior to docking INCA and the diastereomers of INCA-TAME, the compounds were ionized to reflect physiological pH. The compounds were then subjected to energy optimization using the Merck Molecular Force Field (MMFF94). For receptors chosen for docking, the size of docking grids was optimized to recreate the cognate ligand binding mode from the respective X-ray crystal structure. For energy minimization, Autodock 4.2 local search algorithm was applied, starting from the SB-FI 26 binding mode [13, 14]. For *apo*-FABP3, the initial geometry was based on the docking conformation that yielded interactions with Arg107. For applications of biological target prediction software, the compounds were submitted in the simplified molecular-input line-entry system (SMILES) format. UCSF Chimera was used for visualization [15].

## Results

### Binding Affinity of INCA and INCA-TAME with FABPs

**Fig 3:**
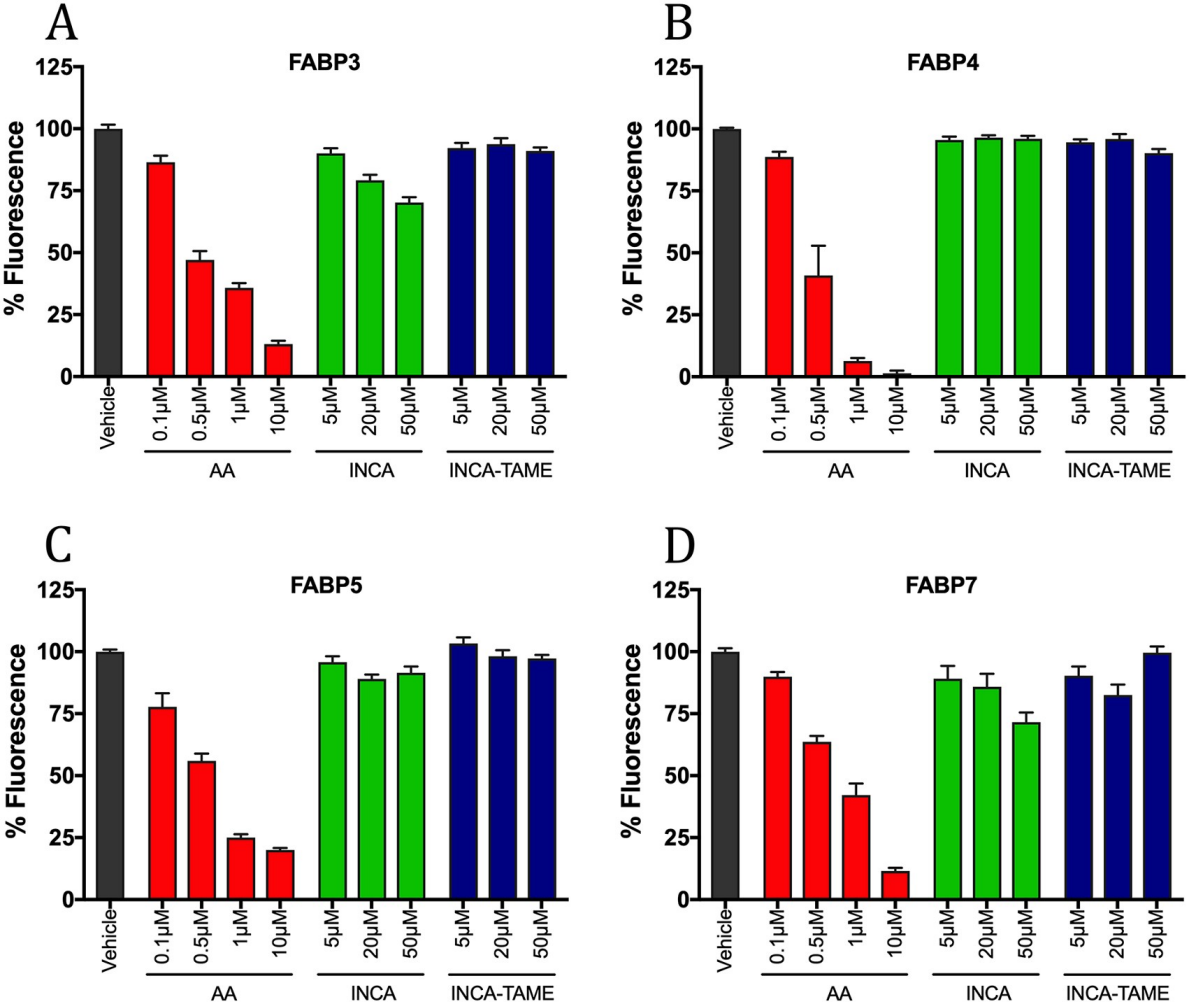
Binding affinity of INCA and INCA-TAME to FABPs. (A) FABP3, (B) FABP4, (C) FABP5, and (D) FABP7 were equilibrated with fluorescent probe and incubated with the indicated concentrations of INCA (green bars), INCA-TAME (blue bars), AA control (red bars), or vehicle (1% DMSO, black bars). Data shown is the mean ± S.E. from at least three independent experiments.

FABP3, FABP5, and FABP7 are expressed in the central nervous system and thus, we assessed whether these FABPs may serve as targets for INCA and INCA-TAME [16] using fluorescent ligand displacement assays. The structures of INCA, INCA-TAME, and the TAME-based FABP5 inhibitor SB-FI-26 are shown in Fig 1. Purified recombinant FABPs were incubated with the fluorescent probe NBD-stearate (FABP3, FABP5, and FABP7) or ANS (FABP4) and fluorescence intensity was monitored to gauge the ability of competitor ligands to displace these probes from the FABP binding pocket. Arachidonic acid (AA), a high-affinity physiological FABP ligand, potently displaced the probe from each of these FABP subtypes (Fig 3). Notably, INCA-TAME displayed no significant affinity for any of the FABP isoforms in the concentration range tested (Fig 3). INCA did not exhibit detectable affinity for FABP4 or FABP5 (Fig 3B and 3C), but very weak binding interactions were observed with FABP3 and FABP7, as evidenced by ~25–30% fluorescence decrease at the highest (50 μM) ligand concentration (Fig 3A and 3D).

### Behavioral effects of INCA and INCA-TAME: Thermal nociception

**Fig 4:**
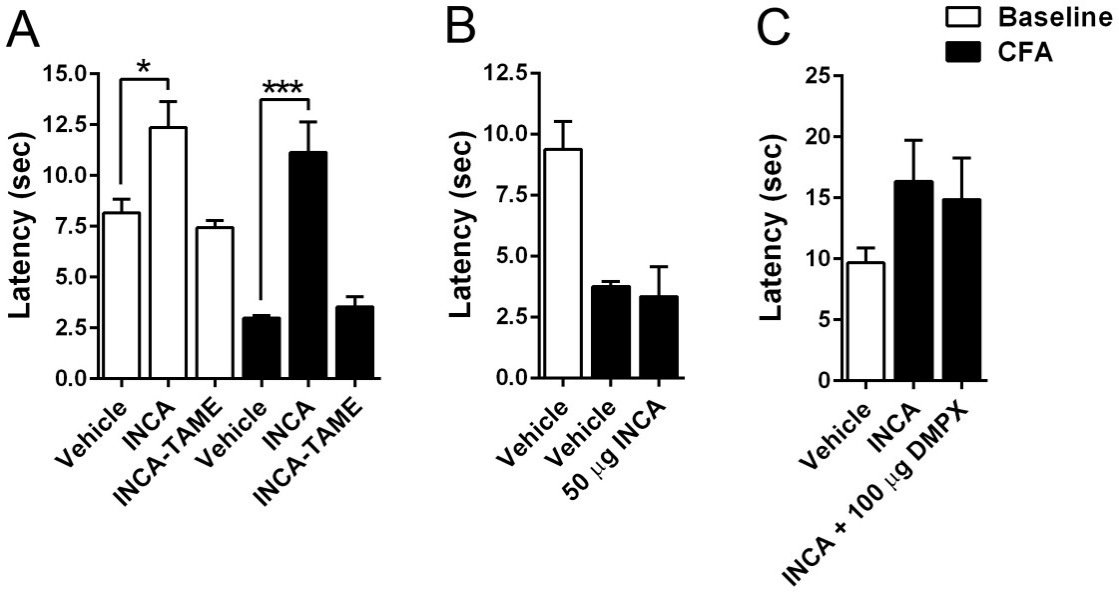
Effect of INCA and INCA-TAME upon thermal hyperalgesia. (A) Thermal withdrawal latencies in male mice at baseline (white bars) and after an intraplantar injection of CFA (black bars). INCA (10 mg/kg, i.p.) and INCA-TAME (20 mg/kg, i.p.) were injected and hindpaw withdrawal latencies were assessed 90 min later. *, p < 0.05; ***, p < 0.001 (n = 6). (B) Thermal withdrawal latencies in mice after intraplantar administration of 50 μg INCA or vehicle (n = 6). (C) Thermal withdrawal latencies in mice receiving 10 mg/kg INCA (i.p.) in the presence or absence of 100 μg DMPX, which was administered via the intraplantar route (n = 6).

The potential antinociceptive effects of INCA-TAME were also assessed in the mouse model of CFA-induced thermal hyperalgesia [17]. Administration of INCA-TAME (20 mg/kg, i.p.) did not affect hind paw withdrawal latencies after CFA-administration (Fig 4A), indicating that this compound does not produce antinociceptive effects. In contrast, TAME-based FABP5 inhibitors produce antinociceptive effects at the same dose [8, 17, 18]. INCA has been reported to produce potent analgesia when administered at doses of 10–20 mg/kg [4, 6]. Indeed, administration of INCA (10 mg/kg, i.p.) produced robust antinociceptive effects in our model (Fig 4A). Importantly, INCA elevated baseline paw withdrawal thresholds (Fig 4A), indicative of a potential sedative effect. This was surprising in light of a previous report demonstrating that INCA does not impair motor function [4].

### Behavioral effects of INCA and INCA-TAME: Locomotion

**Fig 5:**
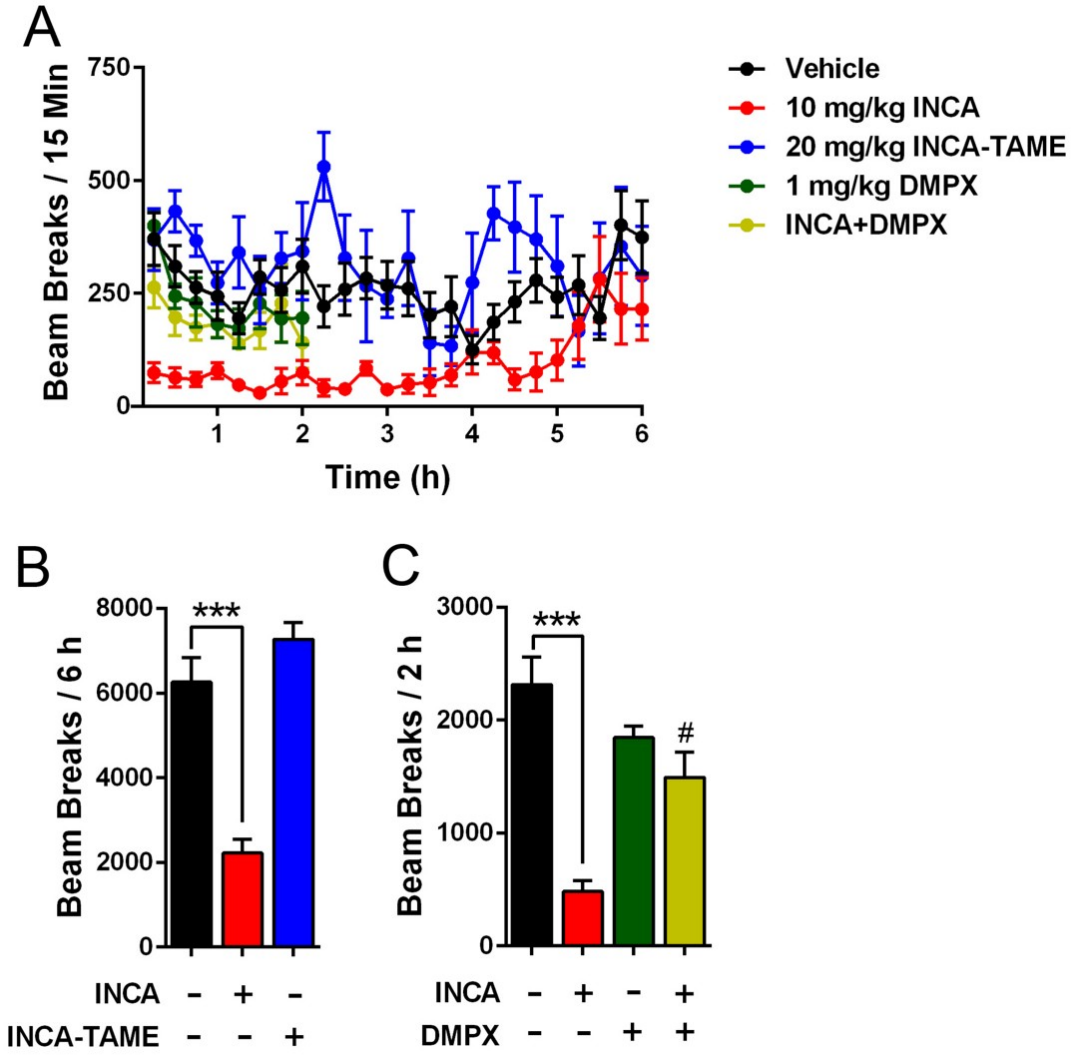
Effects of INCA and INCA-TAME upon homecage activity in mice. (A-C) Locomotor activity in mice injected with vehicle, INCA (10 mg/kg, i.p.), INCA-TAME (20 mg/kg, i.p.), or INCA in the presence or absence of DMPX (1 mg/kg, i.p.). Locomotion was assessed for 2–6 h and inhibitors were injected 15 min prior. ***, p < 0.001; #, p < 0.05 vs INCA (red bar) (n = 8–10).

To determine whether INCA produces motor impairment, we assessed homecage locomotor activity of mice during the dark cycle. Administration of INCA-TAME did not affect home cage activity (Fig 5A and 5B). In contrast, INCA suppressed locomotion, which persisted for ~5 h (Fig 5A and 5B). Previous work indicates that the antinociceptive effects of INCA are mediated by adenosine receptors, principally the adenosine A_1_ receptor [4]. Activation of adenosine A_2_ receptors promotes catalepsy and motor suppression [19–22], and we hypothesized that the motor suppression produced by INCA may be mediated by these receptors. To assess the role of adenosine A_2_ receptors, we employed the adenosine A_2_ receptor antagonist 3,7-Dimethyl-1-propargylxanthine (DMPX) [23, 24]. Since DMPX has an *in vivo* half-life of < 1 h [23], locomotor activity was assessed for 2 h. Administration of DMPX, which had no effect on locomotion when administered alone, reversed the motor suppression induced by INCA (Fig 5C and 5D). Next, we assessed whether the motor suppressive effects of INCA could be uncoupled from its potential antinociceptive effects. Administration of INCA via the intraplantar route did not affect paw withdrawal responses (Fig 4B), indicating that it does not act locally to modulate pain. Similarly, intraplantar administration of DMPX failed to block the effects of systemically administered INCA (Fig 4C). These results indicate that if INCA produces centrally mediated analgesia, such effects are masked by the marked motor suppression induced by INCA.

### Computational analysis of the interactions of INCA and INCA-TAME with FABPs and other potential targets

To gain insight into why INCA does not show experimental activity against FABPs, atomic-level molecular modeling was pursued. The affinities of INCA and two diastereomers of INCA-TAME, i.e., INCA-TAME A and INCA-TAME B, compared to that of SB-FI-26, with FABP3 (PDB: 6AQ1), FABP5 (PDB: 5UR9), and FABP7 (PDB: 5URA) were examined based on their predicted binding geometries (modes) and docking energy scores (Table 1). As Table 1 shows, INCA’s docking energy scores across the three FABPs (-5.93 to -7.55 kcal/mol) are substantially weaker than those of SB-FI-26 (-8.87 to -10.87 kcal/mol), which help support the experimental observations that INCA does not bind to these FABPs.

**Table 1:** Docking energy scores of INCA and INCA-TAME to FABPs.

Compound	FABP5 (PDB: 5UR9) (kcal/mol)	FABP7 (PDB: 5URA) (kcal/mol)	FABP3 (PDB: 6AQ1) (kcal/mol)
**SB-FI 26**	-8.87	-10.87	-10.46
**INCA**	-7.55	-7.32	-5.93
**INCA-TAME A**^*a*^	-6.22	-9.26^*c*^	4.01^*c*^
**INCA-TAME B**^*b*^	-5.75	-10.48^*c*^	-7.69^*c*^

^*a*^ INCA-(*S*,*S*,*S*,*S*)-TAME: 1-[(4*R*,4a*S*,6*R*,7*S*,7a*R*)-2,4,7-trimethyloctahydro-1H-cyclopenta[c]pyridin-6-yl] α-(1*S*,2*S*,3*S*,4*S*)- 2,4-(4-hydroxy-3-methoxyphenyl)-1,3-cyclobutanedicarboxylate

^*b*^ INCA-((*R*,*R*,*R*,*R*))-TAME = 1-[(4*R*,4a*S*,6*R*,7*S*,7a*R*)-2,4,7-trimethyloctahydro-1H-cyclopenta[c]pyridin-6-yl] α-(1*R*,2*R*,3*R*,4*R*)-2,4-(4-hydroxy-3-methoxyphenyl)-1,3-cyclobutanedicarboxylate

^*c*^ No canonical interactions were observed. Thus, this is a non-specific binding, wherein docking does not yield predicted binding poses with canonical electrostatic interactions with the conserved Arg/Tyr diad in FABPs.

**Fig 6:**
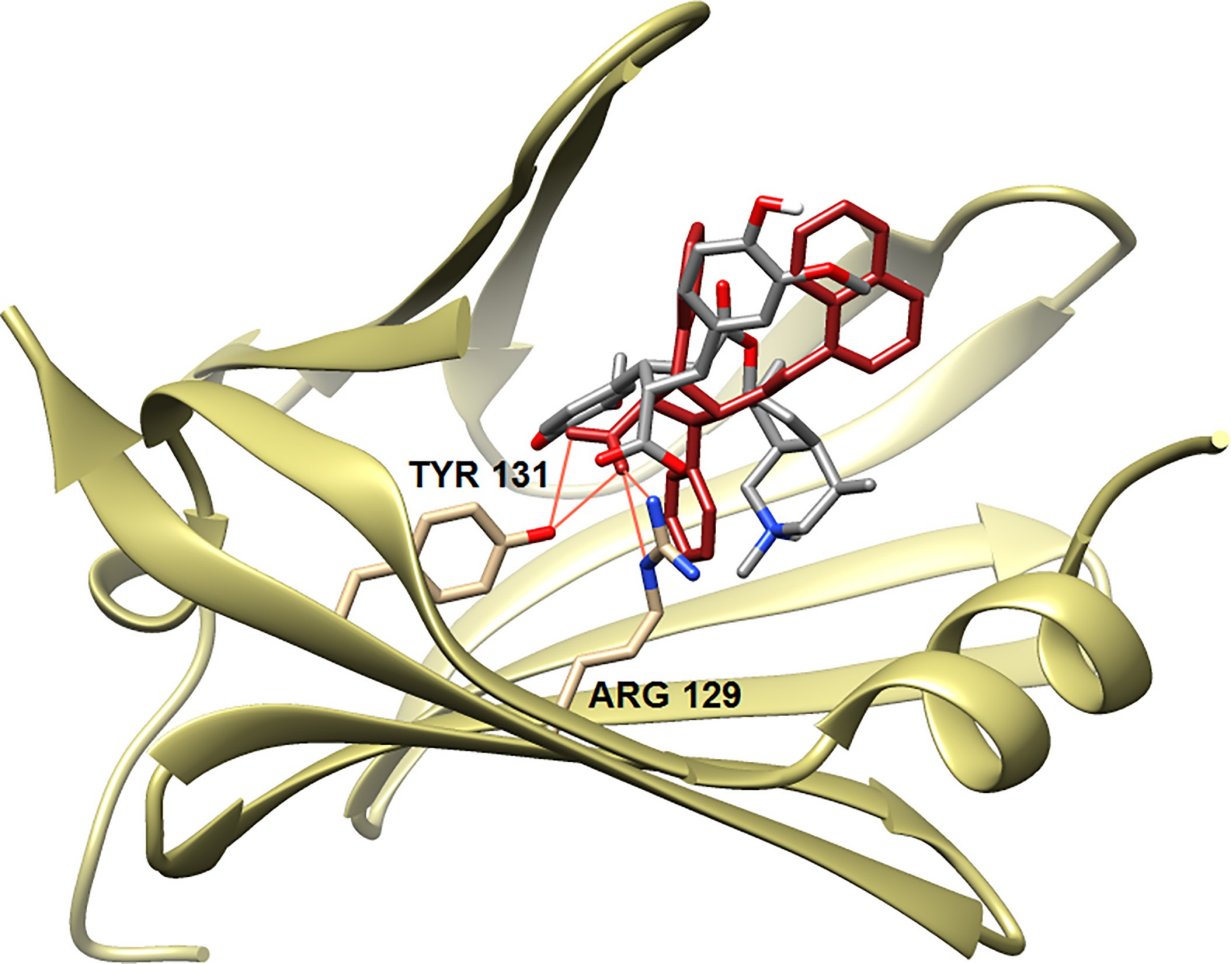
The predicted binding mode of INCA-TAME (gray) in FABP5 (tan) versus the co-crystal structure of SB-FI 26 (red).

The scores for INCA-TAME A and B with FABP5 were also worse than that of SB-FI-26 (or INCA), thus INCA-TAMEs are not expected to bind to FABP5 either. For comparison, Fig 6 compares the predicted binding mode of INCA-TAME A (-6.22 kcal/mol) with the x-ray pose for SB-FI-26 (-8.87) in FABP5. For FABP3, the worse scores for INCA-TAME A and B compared to SB-FI-26 also suggests low affinity for the protein. Although INCA-TAMEs do yield reasonable scores with FABP7, their predicted docking poses do not sustain the critical canonical interactions between the carboxyl group and key amino acid residues, Arg126 and Tyr128 as seen with SB-FI-26. Thus, it is highly unlikely that INCA-TAMEs would bind to FABP7.

Consistent with the docking studies, energy minimizations of INCA-TAME diastereomer A, based on alignments of the SB-FI-26 truxillic core observed in FABP5 (PDB: 5UR9) and FABP7 (PDB: 5URA) x-ray structures [13], also demonstrated dramatically lower predicted affinity (see S1 Table and S1 Text). The minimized geometries were not well accommodated in either FABP site resulting in much lower scores (-5.74 to -6.04 kcal/mol) relative to SB-FI-26 (-8.76 to -10.89 kcal/mol) (S1 Table).

The biological target prediction program SwissTargetPrediction [25] was subsequently applied to search for possible alternative targets for INCA and INCA-TAME. For both INCA and INCA-TAME, it is predicted that ion coupled neurotransmitter transporters are the most likely biological targets, while opioid and cannabinoid receptors are less likely targets (see S2 and S3 Tables, as well as S2 Text). Interestingly, several receptors known to be involved in pain management (i.e. adenosine A_2A_ receptor, PPAR-γ receptor) were not among the predicted targets.

To explore how INCA and INCA-TAME might interact with common receptors for antinociception we performed docking analysis (see S4 Table and S2 Text) starting from crystallographic structures complexed with a cognate ligand for comparison. Overall, compared to cognate ligands, in some cases, the computational analysis predicted that INCA would have fairly good affinity to an adenosine A_2A_ receptor, a PPAR-α receptor, a serotonin receptor and some others (S4 Table). INCA-TAME was also predicted to have fairly good to good affinity with several receptors in a manner similar to that for INCA. However, as described above, INCA-TAME did not show appreciable antinociceptive activity *in vivo*. This marked difference may be attributed to a very high affinity of INCA-TAME with serum albumin, as well as its zwitterionic structure at physiological pH, which is unfavorable for cell membrane permeation.

## Discussion

### INCA and INCA-TAME do not inhibit FABPs

Our group has previously synthesized TAME-based inhibitors targeting FABP5 for the treatment of pain [8, 17]. The structural similarity between the TAME core of FABP5 inhibitors and INCA propelled our interest in assessing whether INCA may exert its analgesic effects wholly or in part through FABP5 inhibition. Our findings demonstrate that INCA does not appreciably interact with any of the brain expressed FABPs (FABP3, FABP5, or FABP7). This is consistent with our previous observations that truxillic acid diesters lacking a free carboxylate demonstrate poor binding to FABPs [17, 18]. We hypothesized that after administration, INCA may be metabolized to INCA-TAME, which possesses a free carboxylate that could increase its affinity for FABPs. However, similar to INCA, INCA-TAME demonstrated poor binding to FABPs, consistent with our recent report that substitutions at the para positions of the phenyl rings of TAMEs compromise binding to FABP5 [17]. This suggests that neither INCA nor its putative metabolite INCA-TAME bind to brain-expressed FABPs, indicating that INCA exerts its physiological effects through a mechanism distinct from FABP inhibition.

### INCA produces motor impairment

The analgesic effects of INCA were originally attributed to its activation of opioid receptors [6]. More recent work indicates that INCA produces analgesia via engagement of adenosine receptors while the contribution of opioid receptors appears to be minimal [4]. While assessing the antinociceptive effects of INCA and INCA-TAME, we observed that administration of INCA produced potent motor suppressive effects as noted by the increased hind paw withdrawal latencies at baseline after treatment with INCA (Fig 4). Consistent with this, homecage activity revealed that INCA, at a dose previously shown to produce analgesia [4, 9], highly suppressed locomotion. Activation of adenosine A_2_ receptors produces catalepsy [19–22] and our results confirmed that the adenosine A_2_ antagonist DMXP reverses the motor suppressive effects of INCA. Our findings contrast previous work which demonstrated that INCA does not alter locomotor activity or induce catalepsy [4], and suggest that INCA produces adenosine receptor-mediated motor impairment at doses that produce analgesia. Given that preclinical assessments of pain require intact motor control, our results suggest that the reported analgesic effects of INCA should be interpreted with caution.

## Supporting information

S1 Table Affinity analysis of INCA-TAME A and SB-FI 26 with FABP3, FABP5, and FABP7 through energy minimization and docking. (PDF)

S2 Table Potential biological targets of INCA based on the SwissTargetPrediction algorithm. (PDF)

S3 Table Potential targets of INCA-TAME based on the SwissTargetPrediction algorithm. (PDF)

S4 Table Docking scores of INCA and INCA-TAME A and B in putative receptors predicted to be involved in antinociception (Autodock Vina). (PDF)

S1 Text Computational affinity analysis of INCA-TAME with FABPs for S1 Table. (PDF)

S2 Text Computer-Predictions of potential biological targets of INCA and INCA-TAME for S2, S3 and S4 Tables. (PDF)

## References

[1] Phillips CJ. The Cost and Burden of Chronic Pain. Rev Pain. 2009;3(1):2–5. 10.1177/204946370900300102. . 26526940 PMC4590036

[2] Butera JA. Current and emerging targets to treat neuropathic pain. J Med Chem. 2007;50(11):2543–6. 10.1021/jm061015w. . 17489576

[3] Gereau RWt, Sluka KA, Maixner W, Savage SR, Price TJ, Murinson BB, et al A pain research agenda for the 21st century. J Pain. 2014;15(12):1203–14. 10.1016/j.jpain.2014.09.004. . 25419990 PMC4664454

[4] Wang ML, Yu G, Yi SP, Zhang FY, Wang ZT, Huang B, et al Antinociceptive effects of incarvillateine, a monoterpene alkaloid from Incarvillea sinensis, and possible involvement of the adenosine system. Scientific reports. 2015;5:16107 10.1038/srep16107. . 26527075 PMC4630779

[5] Chi YM, Nakamura M, Zhao XY, Yoshizawa T, Yan WM, Hashimoto F, et al A monoterpene alkaloid from incarvillea sinensis. Chem Pharm Bull (Tokyo). 2005;53(9):1178–9. . 16141592 10.1248/cpb.53.1178

[6] Nakamura M, Chi YM, Yan WM, Nakasugi Y, Yoshizawa T, Irino N, et al Strong antinociceptive effect of incarvillateine, a novel monoterpene alkaloid from Incarvillea sinensis. J Nat Prod. 1999;62(9):1293–4. . 10514316 10.1021/np990041c

[7] Chi YM, Nakamura M, Yoshizawa T, Zhao XY, Yan WM, Hashimoto F, et al Pharmacological study on the novel antinociceptive agent, a novel monoterpene alkaloid from Incarvillea sinensis. Biol Pharm Bull. 2005;28(10):1989–91. . 16204962 10.1248/bpb.28.1989

[8] Berger WT, Ralph BP, Kaczocha M, Sun J, Balius TE, Rizzo RC, et al Targeting fatty acid binding protein (FABP) anandamide transporters—a novel strategy for development of anti-inflammatory and anti-nociceptive drugs. PLoS One. 2012;7(12):e50968 10.1371/journal.pone.0050968. 23236415 PMC3517626

[9] Huang B, Zhang F, Yu G, Song Y, Wang X, Wang M, et al Gram Scale Syntheses of (-)-Incarvillateine and Its Analogs. Discovery of Potent Analgesics for Neuropathic Pain. J Med Chem. 2016;59(8):3953–63. 10.1021/acs.jmedchem.6b00132. . 27022999

[10] Ichikawa M, Takahashi M, Aoyagi S, Kibayashi C. Total synthesis of (-)-incarvilline, (+)-incarvine C, and (-)-incarvillateine. J Am Chem Soc. 2004;126(50):16553–8. 10.1021/ja0401702. . 15600360

[11] Kaczocha M, Glaser ST, Maher T, Clavin B, Hamilton J, O’Rourke J, et al Fatty acid binding protein deletion suppresses inflammatory pain through endocannabinoid/N-acylethanolamine-dependent mechanisms. Mol Pain. 2015;11:52 10.1186/s12990-015-0056-8. . 26311517 PMC4551694

[12] Luk J, Lu Y, Ackermann A, Peng X, Bogdan D, Puopolo M, et al Contribution of diacylglycerol lipase beta to pain after surgery. Journal of pain research. 2018;11:473–82. 10.2147/JPR.S157208. 29551907 PMC5842774

[13] Hsu H-C, Tong S, Zhou Y, Elmes M, Yan S, Kaczocha M, et al The anti-nociceptive agent SBFI-26 binds to anandamide transporters FABP5 and FABP7 at two different sites. Biochemistry. 2017;56(27):3454–62 10.1021/acs.biochem.7b00194 28632393 PMC5884075

[14] Morris GM, Huey R, Lindstrom W, Sanner MF, Belew RK, Goodsell DS, et al Autodock4 and AutoDockTools4: automated docking with selective receptor flexiblity. J Comput Chem. 2009;16:2785–91 10.1002/jcc.21256 PMC2760638 19399780

[15] Pettersen EF, Goddard TD, Huang CC, Couch GS, Greenblatt DM, Meng EC, et al UCSF Chimera—a visualization system for exploratory research and analysis. J Comput Chem. 2004;25(13):1605–12. 10.1002/jcc.20084. 15264254

[16] Owada Y, Yoshimoto T, Kondo H. Spatio-temporally differential expression of genes for three members of fatty acid binding proteins in developing and mature rat brains. J Chem Neuroanat. 1996;12(2):113–22. https://doi.org/S0891-0618(96)00192-5. . 9115666 10.1016/s0891-0618(96)00192-5

[17] Yan S, Elmes MW, Tong S, Hu K, Awwa M, Teng GYH, et al SAR studies on truxillic acid mono esters as a new class of antinociceptive agents targeting fatty acid binding proteins. Eur J Med Chem. 2018;154:233–52. 10.1016/j.ejmech.2018.04.050. . 29803996 PMC5999033

[18] Kaczocha M, Rebecchi MJ, Ralph BP, Teng YH, Berger WT, Galbavy W, et al Inhibition of fatty acid binding proteins elevates brain anandamide levels and produces analgesia. PLoS One. 2014;9(4):e94200 10.1371/journal.pone.0094200. . 24705380 PMC3976407

[19] Mingote S, Pereira M, Farrar AM, McLaughlin PJ, Salamone JD. Systemic administration of the adenosine A(2A) agonist CGS 21680 induces sedation at doses that suppress lever pressing and food intake. Pharmacol Biochem Behav. 2008;89(3):345–51. 10.1016/j.pbb.2008.01.006. . 18281083 PMC2674372

[20] Hauber W, Munkle M. Stimulation of adenosine A2a receptors in the rat striatum induces catalepsy that is reversed by antagonists of N-methyl-D-aspartate receptors. Neurosci Lett. 1995;196(3):205–8. . 7501284 10.1016/0304-3940(95)11871-s

[21] Hauber W, Munkle M. Motor depressant effects mediated by dopamine D2 and adenosine A2A receptors in the nucleus accumbens and the caudate-putamen. Eur J Pharmacol. 1997;323(2–3):127–31. . 9128830 10.1016/s0014-2999(97)00040-x

[22] Ferre S, Rubio A, Fuxe K. Stimulation of adenosine A2 receptors induces catalepsy. Neurosci Lett. 1991;130(2):162–4. . 1665549 10.1016/0304-3940(91)90387-9

[23] Yang M, Soohoo D, Soelaiman S, Kalla R, Zablocki J, Chu N, et al Characterization of the potency, selectivity, and pharmacokinetic profile for six adenosine A2A receptor antagonists. Naunyn Schmiedebergs Arch Pharmacol. 2007;375(2):133–44. 10.1007/s00210-007-0135-0. . 17310264

[24] Seale TW, Abla KA, Shamim MT, Carney JM, Daly JW. 3,7-Dimethyl-1-propargylxanthine: a potent and selective in vivo antagonist of adenosine analogs. Life Sci. 1988;43(21):1671–84. . 3193854 10.1016/0024-3205(88)90478-x

[25] Gfeller D, Grosdidier A, Wirth M, Daina A, Michielin O, Zoete V. SwissTargetPrediction: a web server for target prediction of bioactive small molecules. Nucleic Acids Res. 2014;42(Web Server issue):W32–8. 10.1093/nar/gku293. . 24792161 PMC4086140

