# Supplementary material for: Incarvillateine produces antinociceptive and motor suppressive effects via adenosine receptor activation
Source: PLoS One. 2019 Jun 25;14(6):e0218619. doi: 10.1371/journal.pone.0218619 (PMC6592529; doi:10.1371/journal.pone.0218619)
Supplement: S1 Table — (PDF) [file pone.0218619.s001.pdf]

S1 Table. Affinity analysis of INCA-TAME A and SB-FI 26 with FABP3, FABP5, and FABP7 through energy minimization and docking

Structure (PDB Code)	SB-FI 26 Energy minimization	SB-FI 26 docking (RMSD to X-ray)	INCA-TAME A[a] Energy minimization	INCA-TAME A[a] docking (RMSD to truxillic acid core of SB-FI-26)
FABP3 (5B28)	---	-10.58	---	-8.37
FABP3 (6AQ1)	---	-10.46	---	4.01[b]
FABP5 (5UR9)	-8.76	-8.87 (0.97 Å)	-6.04	-6.22 (4.11 Å)[b]
FABP7 (5URA)	-10.89	-10.87 (0.7 Å)	-5.74	-9.26 (4.10 Å)[b]

[a]INCA-TAME: 1-[(4*R*,4a*S*,6*R*,7*S*,7a*R*)-2,4,7-trimethyloctahydro-1H-cyclopenta[c]pyridin-6-yl] α-(1*S*,2*S*,3*S*,4*S*)- 2,4-(4-hydroxy-3-methoxyphenyl)-1,3-cyclobutanedicarboxylate.
[b]No canonical interactions observed.
