# Supplementary material for: Incarvillateine produces antinociceptive and motor suppressive effects via adenosine receptor activation
Source: PLoS One. 2019 Jun 25;14(6):e0218619. doi: 10.1371/journal.pone.0218619 (PMC6592529; doi:10.1371/journal.pone.0218619)
Supplement: S2 Table — (PDF) [file pone.0218619.s002.pdf]

S2 Table. Potential biological targets of INCA based on the SwissTargetPrediction algorithm

Protein	Uniprot ID	Number of similar known ligands (2D/3D)
Na-dependent noradrenaline Transporter	P23975	28/1100
Na-dependent serotonin Transporter	P31645	31/891
Na-dependent dopamine Transporter	Q01959	28/1100
Na and Cl dependent glycine Transporter 1	P48067	24/469
Na and Cl dependent glycine transporter 2	Q9Y345	24/469
Na dependent proline transporter	Q99884	24/469
Na and Cl dependent neutral and basic amino acid transporter	Q9UN76	24/469
Renin	P00797	16/176
Cathepsin D	P07339	16/176
Napsin-A	O96009	16/176
Cannabinoid 1 receptor	P21554	5/103
Cannabinoid 2 receptor	P34972	2/99
Mu-type opioid receptor	P41143	21/695
Delta-type opioid receptor	P06276	21/695
