# Supplementary material for: Incarvillateine produces antinociceptive and motor suppressive effects via adenosine receptor activation
Source: PLoS One. 2019 Jun 25;14(6):e0218619. doi: 10.1371/journal.pone.0218619 (PMC6592529; doi:10.1371/journal.pone.0218619)
Supplement: S3 Table — (PDF) [file pone.0218619.s003.pdf]

S3 Table. Potential targets of INCA-TAME based on the SwissTargetPrediction algorithm

Protein Name	Uniprot ID	Number of similar known ligands (2D/3D)
Na-dependent noradrenaline transporter	P23975	2/1100
Na-dependent serotonin transporter	P31645	2/891
Na- and Cl-dependent glycine transporter 1	P48067	38/469
Na-dependent dopamine transporter	Q01959	2/1100
Na-dependent proline transporter	Q99884	38/469
Na- and Cl-dependent neutral and basic amino acid transporter	Q9UN76	38/469
Na- and Cl-dependent glycine transporter 2	Q9Y345	38/469
Renin	P00797	32/176
Cathepsin D	P07339	32/176
Napsin-A	O96009	32/176
Mu-type opioid receptor	P41143	338/695
Delta-type opioid receptor	P06276	338/695
Kappa-type opioid receptor	P41145	153/529
Neprilysin	P08473	87/97
Membrane metallo-endopeptidase like 1	Q495T6	87/97
