# Supplementary material for: Incarvillateine produces antinociceptive and motor suppressive effects via adenosine receptor activation
Source: PLoS One. 2019 Jun 25;14(6):e0218619. doi: 10.1371/journal.pone.0218619 (PMC6592529; doi:10.1371/journal.pone.0218619)
Supplement: S4 Table — (PDF) [file pone.0218619.s004.pdf]

S4 Table. Docking scores of INCA and INCA-TAME A and B in putative receptors predicted to be involved in antinociception (Autodock Vina).

	PDB Code	Cognate Ligand Score (kcal/mol)	INCA Score (kcal/mol)	INCA-TAME A Score (kcal/mol)	INCA-TAME B Score (kcal/mol)
Adenosine receptors A_{2A}	4UG2	-10.9	-6.7	-6.6	-6.9
A_{2A} (agonist)	3QAK	-10.6	-7.4	-7.9	-7.4
A_{2A} (soluble cytochrome b562)	5OLO	-9.8	-9	-8.7	-8.7
A_{2A} (agonist)	5WF5	-11	-7.5	-8	-8.6
Cannabinoid 1 receptors	5UO9	-12.3	-8.2	-7.4	-8.2
	5XR8	-11.7	-2.5	-9.2	-9.4
PPAR-γ receptors	2OM9	-9.3	-10.3	-10	-9.4
	4XUM	-9.2	-6.4	-7.9	-8.2
Serotonin receptors	5TVN	-9.4	-4.6	-8	-9.4
(methiothepin)	5V54	-9.8	-9.1	-9	-9.4
(ergotamine)	6BQG	-14.2	-10.4	-10.1	-10.8
Sodium-dependent serotonin receptor	6AWO	-9.2	-6.3	-8.9	-9.7
Mu-type opioid receptor	5C1M	-12.1	-6.2	-9.3	-8.5
Kappa-type opioid receptor	4DJH	-10.9	-9.5	-8.5	-9.1
Delta-type opioid receptor	4EJ4	-11.1	-7.7	-8.4	-9.2
Calcium ion channel	5EK0	-11.6	-8.2	-8	-8
TRPV1 ion channel	6BWJ	-9.4	-8	-6.8	-7.7
TNF-α	5MU8	-9.4	-8.5	-8	-8.7
