# Supplementary material for: Incarvillateine produces antinociceptive and motor suppressive effects via adenosine receptor activation
Source: PLoS One. 2019 Jun 25;14(6):e0218619. doi: 10.1371/journal.pone.0218619 (PMC6592529; doi:10.1371/journal.pone.0218619)
Supplement: S1 Text — (PDF) [file pone.0218619.s005.pdf]

S1 Text. Computational affinity analysis of INCA-TAME with FABPs for S1 Table

The computational analysis of INCA-TAME in FABPs proceeded from the X-ray structure of SB-FI 26 in FABP5 and FABP7 (S1 Table). Since only the (*S,S,S,S*)-enantiomer of SB-FI 26 was crystallized only INCA-TAME A was analyzed. For FABP3, the analysis was based on docking to a complex co-crystallized with palmitic acid. Docking and energy minimization scores are shown in Table S1 along with RMSDs of the common truxillic acid core for comparison. As controls, SB-FI 26 were redocked into FABP5 and FABP7. The low RMSDs and similarity in scores for both FABP5 (0.97 Å, -8.87 vs -8.76 kcal/mol docked vs x-ray) and FABP7 (0.7 Å, -10.87 vs -10.89 kcal/mol, docked vs x-ray) indicates good accuracy of the docking protocols.

The INCA-TAME A energy minimizations, starting from the SB-FI 26 binding pose, led to much lower scores (-5.75 and -6.04 kcal/mol) relative to SB-FI 26 (-8.76 and -10.89 kcal/mol) which indicates the compounds are not well-accommodated in either modeled binding site and thus may not bind FABP5 or FABP7. Interestingly, for FABP5, the docking and minimization scores were very similar (-6.22 and -6.04 kcal/mol) and the compound was correctly predicted to have poor affinity to FABP5. For FABP7, however, docking and minimization scores showed a large energy difference of 3.52 kcal/mol suggesting an alternative binding mode. In both cases however, docking appeared to shift the INCA-TAME truxillic acid core in by ~ 4 angstroms indicating that the SB- FI26 canonical pose was not as energetically viable. With respect to the analysis in FABP3 (crystal structures 5B28 and 6AQ1), the docking scores correctly predicted lower affinity for INCA-TAME A relative to SB-FI-26.
