# Supplementary material for: Incarvillateine produces antinociceptive and motor suppressive effects via adenosine receptor activation
Source: PLoS One. 2019 Jun 25;14(6):e0218619. doi: 10.1371/journal.pone.0218619 (PMC6592529; doi:10.1371/journal.pone.0218619)
Supplement: S2 Text — (PDF) [file pone.0218619.s006.pdf]

S2 Text. Computer-Predictions of potential biological targets of INCA and INCA-TAME for S2, S3 and S4 Tables

Since INCA does not appear to mediate anti-nociception through FABP inhibition, other potential biological mechanisms were explored *in silico.* The computational analysis of INCA began by applying the Swiss Target Prediction (www.swisstargetprediction.ch) [1] to get an idea of potential targets of the alkaloid compound aside from the "classic" anti-nociception targets. These results are shown in S2 Table and S3 Table. Targets are listed in order of decreasing probability based on the algorithms.

What is striking amongst the list of predicted targets for INCA (S2 Table) and INCA-TAME (S3 Table) are the multiple anti-nociception relevant targets listed. The absence of FABPs as targets is consistent with other *in silico* and *in vitro* data. Interestingly, ion-coupled neurotransmitter transporters ranked highest among the predicted targets of INCA rather than either opioid receptors or adenosine receptors. Noradrenaline reuptake inhibition, the target calculated as the most probable by the SwissTargetPrediction algorithm, is a known mechanism of pain relief based on anti-depressant efficacy in certain types of neuropathic pain. Nevertheless, opioid receptors also emerge as less likely predicted targets of INCA and INCA-TAME.

Although the SwissTargetPrediction algorithm did identify several anti-nociception relevant targets there was also an absence of several common receptors known to be involved in pain management. This prompted us to perform docking analysis of INCA and INCA-TAME against known putative targets involved in antinociception, including adenosine receptors. The results are summarized in S4 Table.

The docking analysis predicts that INCA does not have biologically relevant level of affinity to CBI receptors and three out of four adenosine A2 receptors examined. (*Note: In this discussion, "biologically relevant level of affinity" is defined to be <1.5 kcal/mol energy score difference from that observed for the cognate ligand since absolute docking scores across different receptors have little significance.*) Nevertheless, INCA shows relatively good affinity to one adenosine A_{2A} receptor (pdb: 5OLO). This prediction is consistent with the result shown in Figure 5 wherein the motor suppressive effect of INCA was reversed by the adenosine A_2 antagonist DMXP. It is also predicted that INCA has a very good affinity to a one PPAR-γ receptor (pdb: 2OM9) and one serotonin receptor (pdb: 5V54). Accordingly, INCA may bind to these representative receptors involved in antinociception, as well as adenosine A_2 receptors unselectively. It is also predicted that INCA has fairly good affinity to a kappa opioid receptor (pdb: 4DJH), a TRPV1 ion channel receptor (pdb: 6BWJ) and a TNF-α receptor (pdb: 5MU8).

It should be noted that INCA-TAMEs (A and/or B diastereomer) are predicted to have fairly good or good affinity to one PPAR-γ receptor (pdb: 2OM9), two serotonin receptors (pdbs: 5TVN, 5V54), a sodium-dependent serotonin receptor (pdb: 6AWO) and TNF-α (pdb: 5MU8). However, INCA-TAME did not show appreciable antinociceptive effect *in vivo*. This marked contrast to INCA is likely to be attributed to its strong binding to serum albumin. Also, INCA-TAME is an amino acid and may well be zwitterionic at physiological pH, which is unfavorable for cell membrane permeation.

1. Gfeller D, Grosdidier A, Wirth M, Daina A, Michielin O, Zoete V. wissTargetPrediction: a web server for target prediction of bioactive small molecules. Nucleic Acids Res. 2014;42:W32-8. doi: 10.1093/nar/gku293.
